# Microbial Secondary Metabolites via Fermentation Approaches for Dietary Supplementation Formulations

**DOI:** 10.3390/molecules28166020

**Published:** 2023-08-11

**Authors:** Alexandru Vasile Rusu, Monica Trif, João Miguel Rocha

**Affiliations:** 1 CENCIRA Agrofood Research and Innovation Centre, Ion Meșter 6, 400650 Cluj-Napoca, Romania; rusu_alexandru@hotmail.com; 2 Food Research Department, Centre for Innovative Process Engineering (CENTIV) GmbH, 28857 Syke, Germany; 3 Universidade Católica Portuguesa, CBQF—Centro de Biotecnologia e Química Fina, Laboratório Associado, Escola Superior de Biotecnologia, Rua Diogo Botelho 1327, 4169-005 Porto, Portugal; 4 LEPABE—Laboratory for Process Engineering, Environment, Biotechnology and Energy, Faculty of Engineering, University of Porto, Rua Dr. Roberto Frias, s/n, 4200-465 Porto, Portugal; 5 ALiCE—Associate Laboratory in Chemical Engineering, Faculty of Engineering, University of Porto, Rua Dr. Roberto Frias, s/n, 4200-465 Porto, Portugal

**Keywords:** dietary supplement, microbial metabolite, white biotechnology, genetic engineering, microorganism

## Abstract

Food supplementation formulations refer to products that are designed to provide additional nutrients to the diet. Vitamins, dietary fibers, minerals and other functional compounds (such as antioxidants) are concentrated in dietary supplements. Specific amounts of dietary compounds are given to the body through food supplements, and these include as well so-called non-essential compounds such as secondary plant bioactive components or microbial natural products in addition to nutrients in the narrower sense. A significant social challenge represents how to moderately use the natural resources in light of the growing world population. In terms of economic production of (especially natural) bioactive molecules, ways of white biotechnology production with various microorganisms have recently been intensively explored. In the current review other relevant dietary supplements and natural substances (e.g., vitamins, amino acids, antioxidants) used in production of dietary supplements formulations and their microbial natural production via fermentative biotechnological approaches are briefly reviewed. Biotechnology plays a crucial role in optimizing fermentation conditions to maximize the yield and quality of the target compounds. Advantages of microbial production include the ability to use renewable feedstocks, high production yields, and the potential for cost-effective large-scale production. Additionally, it can be more environmentally friendly compared to chemical synthesis, as it reduces the reliance on petrochemicals and minimizes waste generation. Educating consumers about the benefits, safety, and production methods of microbial products in general is crucial. Providing clear and accurate information about the science behind microbial production can help address any concerns or misconceptions consumers may have.

## 1. Introduction

In modern society, the subject of health is becoming more and more significant. People’s diets can have both positive and negative effects on their health. The primary goal of nutrition is to provide the body with all the nutrients it needs in order to prevent deficits and promote optimal function [1,2]. Surprisingly, consumers spend over a billion euros annually on dietary supplements. They include substances that are concentrated and meant to have a nutritional or physiological effect, and might be added to a regular diet but don’t have any medical properties. However, most consumers do not actually need to take such supplements [3,4]. Foods that are designed to have an extended and focused impact on physiological parameters in the consumer beyond their nutritional role are increasingly referred to as functional foods. Functional foods offer a positive additional health benefit or are meant to minimize the risk of disease in addition to their nutritional value [5,6].

In foods are incorporated certain substances (e.g., vitamins, minerals, bacterial cultures, unsaturated fatty acids) to positively influence one or more body functions. In contrast to food supplements, functional foods are introduced to the market in the shape of regular foods [7,8,9,10,11]. Sometimes, the term “functional foods” is interchanged with terms like “designer foods” and “nutraceuticals”. Legal definitions for “functional foods” are lacking. As a result, they are available for example on the German market as both nutritional goods, such like margarine enhanced with plant sterols, and foods for general consumption like probiotic yogurt [12,13]. To boost health, soft drinks, milk and dairy products, cereal based products, and spreadable fats are frequently fortified with additional substances. Other examples of useful functional foods include yogurts containing probiotic lactic acid bacteria, vitamin-enriched fruit juices, or omega-3 fatty acid-enriched bread [14,15,16,17].

A recent study found that the European market for food supplements was valued at USD 14.95 billion in 2019 and projected to reach USD 33.80 billion by 2027 [18]. Despite the fact that the spectrum of these foods has expanded significantly in recent years, scientific studies have been conducted to demonstrate the effectiveness of the foods’ functions developed. In principle, “functional foods” that fall under the definition of novel foods under the Novel Foods Regulation or that contain novel ingredients cannot be freely marketed; instead, they must go through a European clearance process. Since 2007, the European Health Claims Directive has required manufacturers to present scientific proof for the added health benefits of the ingredients from products [19,20].

Vitamins, dietary fibers, minerals, and other functional compounds are concentrated in supplements, and are frequently sold in pharmacies or health food stores as tablets, capsules, or liquids. Even though they have some similarities to medications, from a legal standpoint they are considered foodstuffs and, unlike medications, are not required to get a marketing authorization [21,22]. Despite the fact that food supplements (also known as dietary supplements) are foodstuffs rather than medicines, pharmacies are offering and selling a wider variety of these substances. The variety of items available is as extensive and varied as their nutritional-physiological value. Even for specialists, it can be challenging and difficult to get an overview of the scope of these products’ diversity and abundance [23,24,25].

Food supplementation formulations can be used to address specific nutritional deficiencies, support overall health and well-being, or cater to specific dietary needs. Nutrient deficiencies often happen during specific stages of life. It is important that while food supplementation formulations can be beneficial in certain cases, they should not replace a balanced diet. It is generally recommended to consult with a healthcare professional or registered dietitian before starting any new supplementation regime to ensure it aligns with individual needs and health goals. Both the onset and progression of diseases that are impacted by nutrition should be avoided [26]. It must be avoided conveying the idea that “food supplements” are required because a balanced, diverse diet would not deliver enough of certain essential nutrients [27]. Some consumers believe that taking dietary supplements or eating functional foods on occasion might mitigate the risk of an unhealthy diet. This hypothesis is not the case, and switching to a permanent healthy diet is both healthier and less expensive. Therefore, it must be made clear that food supplements should not be utilized in place of a diversified diet [28,29]. Specific amounts of dietary elements are given to the body through supplements, and these include so-called non-essential compounds such as secondary plant bioactive components or microbial natural products in addition to nutrients in the narrower sense [30,31,32].

**Figure 1:**
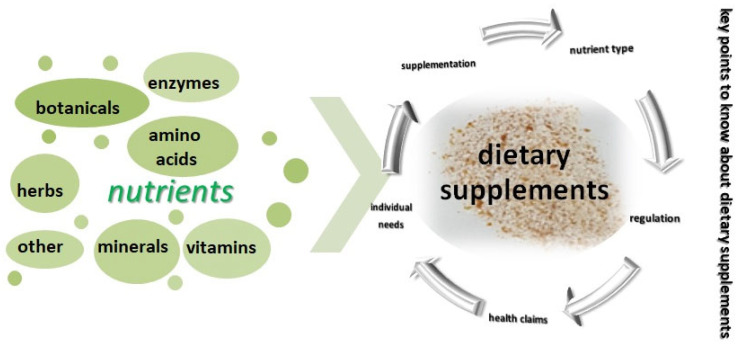
Key points to know about dietary supplements.

Dietary supplements contain different nutrients, such as vitamins, minerals, herbs, or other botanicals, amino acids, enzymes, or other substances, intended to supplement the diet (Figure 1), and can be incorporated in various final products forms, as already mentioned, including pills, capsules, tablets, liquids, powders, and even energy bars.

There are some key aspects to keep in mind about dietary supplements [33,34,35]:

- Supplementation: Dietary supplements are meant to complement a person’s diet when it may be difficult to obtain all necessary nutrients through food alone. They aim to fill nutritional gaps and provide additional nutrients that may be lacking;
- Nutrient types: Common nutrients found in dietary supplements include vitamins (such as vitamin C, vitamin D, or B-complex vitamins), minerals (like calcium, iron, or magnesium), and herbal or botanical extracts (such as ginkgo biloba or Echinacea);
- Regulation: In many countries, dietary supplements are regulated as a category of food, rather than drugs. Regulations vary, but typically supplements must be labeled accurately and must not make false or misleading claims about their benefits;
- Health claims: Dietary supplements are often marketed with various health claims, but it is important to approach these claims with skepticism. While some supplements have been studied extensively and may have proven benefits, others may have limited or no scientific evidence to support their claimed effects. The safety and quality of dietary supplements can vary. It is important to choose reputable brands that adhere to good manufacturing practices. Additionally, some supplements may interact with medications or have adverse effects, so it’s wise to consult a healthcare professional before starting any new supplement regimen;
- Individual needs: Not all individuals require dietary supplements. In general, it is best to obtain nutrients through a balanced diet rich in fruits, vegetables, whole grains, lean proteins, and healthy fats. However, certain population groups, such as pregnant women, older adults, or individuals with specific dietary restrictions, may benefit from targeted supplementation under the guidance of a healthcare professional.

Common types of food supplementation formulations are listed below [36,37,38,39,40,41]:

(I) Multivitamins: These formulations contain a combination of essential vitamins and minerals to support general health; their aim is to provide a comprehensive range of nutrients that may be lacking in a person’s diet.
(II) Omega-3 fatty acids: Omega-3 supplements often come in the form of fish oil or algae oil capsules. They provide essential fatty acids, such as eicosapentaenoic acid (EPA) and docosahexaenoic acid (DHA), which are beneficial for heart health, brain function, and inflammation reduction.
(III) Protein supplements: These formulations are popular among athletes, bodybuilders, and individuals who may have increased protein needs. Protein supplements often come in powder form, are made from sources such as whey, casein, soy, or pea protein, and can be used to support muscle growth, recovery, and overall protein intake.
(IV) Calcium and vitamin D: These supplements are commonly taken to support bone health. Calcium is essential for strong bones, while vitamin D aids in calcium absorption.
(V) Iron supplements: Iron is crucial for the production of red blood cells and oxygen transport in the body. Iron supplementation is often recommended for individuals with iron deficiency or increased iron needs, such as pregnant women or those with certain medical conditions.
(VI) Probiotics: Probiotic supplements contain beneficial bacteria that support a healthy gut microbiome. They can help improve digestion, boost immune function, and promote overall gut health.
(VII) Herbal supplements: These formulations contain various plant extracts or botanical ingredients. Herbal supplements are often used to support specific health goals or address certain conditions, but their effectiveness and safety may vary.

Even if it is not always obvious, biotechnology is becoming increasingly vital in everyday life. Biotechnology is used in the production of many products, such as the homemade, meals, medications, and chemicals. These include a variety of commodities, such as bread, cheese, beer, and wine, for which people have relied on the power of live microorganisms for ages [42,43,44].

**Figure 2:**
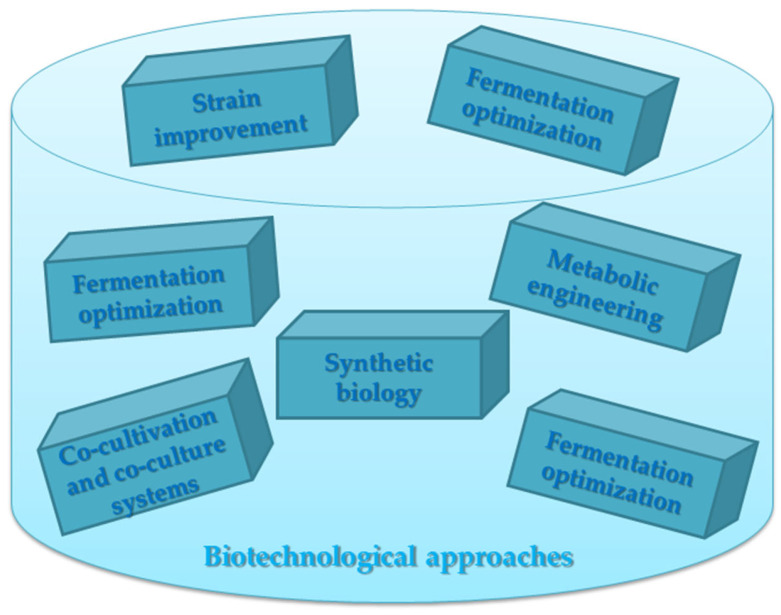
Biotechnological approaches to produce microbial secondary metabolites.

Biotechnological approaches are increasingly being used to produce microbial secondary metabolites (Figure 2) [45,46,47,48,49,50,51,52,53,54,55,56,57,58,59,60]:

Strain improvement: Microbial strains can be genetically modified or selected through breeding techniques to enhance the production of desired secondary metabolites. This can involve techniques such as mutagenesis, recombinant DNA technology, or genome editing to manipulate the metabolic pathways of microorganisms.

Fermentation optimization: Fermentation processes can be optimized to maximize the production of secondary metabolites. This includes optimizing the culture conditions such as temperature, pH, oxygen levels, and nutrient availability. Additionally, fed-batch or continuous fermentation strategies can be employed to enhance productivity.

Metabolic engineering: Metabolic engineering involves manipulating the metabolic pathways of microorganisms to redirect their metabolic flux towards the production of specific secondary metabolites. This can be achieved by overexpressing key enzymes; deleting or downregulating competing pathways; or introducing genes from other organisms to enhance productivity.

Synthetic biology: Synthetic biology techniques enable the design and construction of novel genetic circuits in microorganisms to produce specific secondary metabolites. This approach involves combining genetic parts, such as promoters, regulatory elements, and enzymes, to create new metabolic pathways or enhance existing ones.

Co-cultivation and co-culture systems: Co-cultivation involves growing multiple microbial strains together to take advantage of their synergistic interactions for secondary metabolite production. This approach allows for the exchange of metabolites, signaling molecules, or co-factor regeneration between different strains, leading to increased yields or the production of new metabolites.

Heterologous expression: Heterologous expression involves transferring the genes responsible for secondary metabolite production from one microorganism to another host organism, such as bacteria or yeast, which can be more amenable to large-scale production. This approach allows for the production of secondary metabolites that are not naturally produced by the host organism.

Genome mining and bioinformatics: Genome mining involves the identification of potential secondary metabolite gene clusters in the genomes of microorganisms using bioinformatics tools. This approach helps in the discovery of new compounds and facilitates the characterization of the biosynthetic pathways involved in their production.

These biotechnological approaches offer great potential for the production of microbial secondary metabolites. They allow for the optimization and scale-up of production processes, as well as the discovery and production of new bioactive compounds with various applications in medicine, agriculture, and industry.

The objective of the current review is to briefly summarize other relevant dietary supplements and natural substances (e.g., vitamins, amino acids, antioxidants) used in the production of dietary supplements formulations and their microbial natural production via fermentative biotechnological approaches.

## 2. Biotechnological Production’ Role in the Context of Sustainable Microbial Natural Products

Biotechnology plays a significant role in the development and production of microbial-sourced dietary supplementation formulations. Microbial-sourced dietary supplements typically involve the use of microorganisms, such as bacteria, yeast, or fungi, to produce beneficial compounds that can be used to enhance human health or nutrition. Biotechnology plays a vital role in harnessing the capabilities of microorganisms to produce beneficial compounds for dietary supplementation. It enables the development of more efficient, scalable, and safe processes for the production of microbial-sourced dietary supplements, contributing to advancements in the field of nutritional and functional food products.

White biotechnology approaches have become an integral part of manufacturing processes in the creation of high-quality in different industries and applications such as chemicals, medications, vitamins, detergents and cleaning agents, textile, leather, and paper finishing, and the production of many other common products. White biotechnology, well-known as industrial biotechnology, is a subfield of biotechnology. It is the application of scientific and technological knowledge to living microorganisms and their products [45,46,47].

Living microorganisms have been employed to make cheese, sourdough bread, and wine, and produce an alcoholic beer-like drink from germinated barley since the dawn of civilization. Long before the discovery of microorganisms or the comprehension of the underlying mechanisms, many cultures had developed techniques for fermenting sweet foods into alcohol using yeasts, or vinegar manufacture [48]. Only in the past three centuries microorganisms and the metabolic underpinnings of fermentation processes have been discovered [49,50]. Pasteur’s experiments laid the groundwork for understanding fermentation and current microbiology. His discovery that “the role of the infinitely small in nature is infinitely large” helped pave the road for modern biotechnology.

On the other hand, modern biotechnology is distinguished primarily by its specific application of molecular biology techniques. This led to the development of genetic engineering, which is today a crucial component of biotechnology because it gives biotechnologists the resources that they need to do their jobs thanks to the decoded molecular blueprints of living things [51]. While genetic engineering is often considered to refer to the examination and precise manipulation of an organism’s or cell’s genetic code, biotechnological processes go beyond this and integrate technical elements with cell and molecular biology, as in the case of bioprocess engineering. Organisms or specific biomolecules are employed as the foundation for industrial production in industrial biotechnology [52,53]. This makes it to be different than red (medical-pharmaceutical biotechnology) biotechnology and green (agricultural-plant biotechnology) biotechnology. When molecular biology and genetics were discovered in the latter half of the 20th century, the real scientific revolution got underway. Bioinformatics, and genome analysis in particular, have become indispensable, not only to identify the urgently needed new classes of substances, but also to fathom the molecular details of their modes of action [54].

The ability to systematically harness the productive capacities of microorganisms and cells and to analyze each of their distinct genetic components at the genetic level was only made possible by advancements in genome research. It fueled the rapid advancement of contemporary biotechnology. This is especially true in light of the identification of DNA as a hereditary molecule and the potential for targeted genetic modification [55,56]. The evolutionary metabolic variety of living nature could now be particularly utilized for industrial processes thanks to this knowledge. Science will then be able to comprehend how nature creates diversity and how this diversity may be applied to procedures employed in industrial production [57].

The resources found in nature have drawn more and more of society’s attention as a result of the need for a sustainable economy. Politic initiatives and industry sector have come to realize that current industrial practices cannot guarantee the long-term preservation of natural resources for future generations Above all, the limited availability of fossil fuels and other basic materials prompted reconsideration and accelerated the search for substitutes [58]. Since then, microorganisms create a rich but not fully explored reservoir of several natural compounds with a broad spectrum of biological. The limitless diversity of nature has just recently been understood, which is one of the reasons why the potential is so immense [59]. Scientists search every imaginable ecosystem of potential applications, including soils from all over the world, water, and sediments from the deep sea to desert sands. There are a variety of microorganisms that number anywhere between 100 million and more, according to scientists. Only 1% of them can now be distinguished by microbiologists. To gain an understanding of the enormous diversity, scientists have become experts in analyzing and deciphering microbial genomes. There are now hundreds of decoded microbe genomes available, but the potential remains far from being fully explored given the thousands of described and undiscovered microorganisms [60,61].

Microbial natural products have gained significant attention in recent years due to their potential applications as supplements. These products are derived from microorganisms such as bacteria, fungi, actinomycetes, and so-called secondary metabolites. Secondary metabolites are not essential for microbial growth but are typically produced during specific growth phases or in response to environmental stimuli. Secondary metabolites have diverse chemical structures and functions; many of them have demonstrated potential benefits for human health, making them valuable sources of bioactive compounds for various applications, including food supplements and functional foods [54,59].

For instance, some secondary metabolites produced by microbes have antimicrobial properties, which can help combat pathogenic bacteria and fungi [61]. Other secondary metabolites, such as certain polyphenols, flavonoids, and alkaloids, have been found to possess antioxidant, anti-inflammatory, and anticancer activities. Additionally, microbial metabolites can interact with the human immune system and modulate immune responses. They can influence the development and regulation of the immune system, potentially impacting immune-related diseases and disorders [62,63].

Microorganisms can be genetically engineered to produce alternative proteins that mimic the taste and texture of animal-based proteins. These proteins, such as those derived from bacteria or fungi, can serve as plant-based alternatives in various food products, including meat substitutes, dairy alternatives, and protein supplements. Another application of biotechnology in microbial-sourced dietary products is the production of enzymes. Microorganisms can be engineered to produce specific enzymes that aid in food processing and digestion. Moreover, biotechnology enables the development of microbial strains with enhanced nutritional profiles. Microbes can be genetically modified to produce higher levels of certain nutrients, such as vitamins, minerals, or essential amino acids. These nutrient-enriched microbial strains can be used as food additives or functional ingredients to improve the nutritional content of various dietary products.

Biotechnology offers valuable tools for the development and production of microbial-sourced dietary products. It enables the creation of novel ingredients, enhances nutritional profiles, improves food processing, and contributes to the growth of the alternative protein market. These advancements in biotechnology have the potential to provide sustainable and nutritious options for consumers, while reducing the environmental impact associated with traditional food production methods. Research into microbial metabolites and their effects on human health is an active and expanding field. While many potential benefits have been identified, further studies are needed to fully understand the mechanisms of action and to explore their potential therapeutic applications. In the current review other relevant dietary supplements (rather than protein, carbohydrates, etc.) used in production of dietary supplements and microbial natural produced via fermentative biotechnological approaches are reviewed.

## 3. Microbial Natural Products via Fermentation and Their Potential Applications for Dietary Supplements Formulations

White (industrial) biotechnology is a crucial pillar of the bioeconomy, which is a system of sustainable management based on biological resources [58,64].

The fact that biotechnological procedures can frequently be carried out under moderate, ecologically acceptable circumstances gives them an advantage over chemical processes. While chemical reactions require high temperatures and pressures, microorganisms may change complicated substances with high yields at ambient temperature and normal pressure. Therefore, industrial biotechnology is always associated with ecological expectations, many of which have already been met. However, advancements have recently started in many other application areas, particularly in the manufacturing of products from renewable raw materials [65]. Although they are usually invisible to the naked eye, bacteria, fungi, and viruses play a crucial role in life on our planet. In the global cycle of carbon and nitrogen, microorganisms are irreplaceable. Many examples show how much dependency on microorganisms is in our everyday lives and also in industry. Since only a fraction of the microorganisms on Earth are known so far, research into “microbial dark matter” holds great potential for new discoveries and their use by humans. Microorganisms play a central role in nature’s material cycle, and the increased use of microorganisms also offers great opportunities in the establishment of a sustainable, bio-based economy [66,67]. Biotechnology plays a crucial role in optimizing fermentation conditions to maximize the yield and quality of the target compounds. Factors such as temperature, pH, oxygen availability, nutrient composition, and process scale-up are carefully controlled to achieve optimal product formation. Vitamins, minerals, essential amino acids (and especially branched-chain amino acids, or BCAAs), enzymes, or plant extracts are just a few of the numerous nutrition supplements available on the market (Figure 1). These essential metabolites are needed for microbial growth, reproduction, and basic cellular functions are crucial for the survival and growth of microorganisms, and some of them also have direct benefits for human health and are often obtained through the diet or microbial synthesis in the gut. Each of them has a unique health benefit, but however, there is currently little evidence to support the majority of these supplements’ efficacy [68,69,70].

### 3.1. Microbial Production of Essential Amino Acids

Amino acids are one of a large family of products that are predominantly produced by various types of microorganism such as bacteria. They are the building blocks of proteins, which make up a large part of almost all cells and accelerate or catalyze the biochemical processes of life. Proteins consist of 21 different amino acids, of which humans and animals can only produce 12 themselves [40,71]. The remaining nine essential amino acids (histidine, isoleucine, leucine, lysine, methionine, phenylalanine, threonine, tryptophan and valine) must be ingested with food [72]. Biotechnologically produced amino acids play an important role in the direct administration of nutrients into the bloodstream, i.e., bypassing the intestine, the so-called parenteral nutrition of humans. They are also important in animal nutrition [40]. Amino acid demand is increasing not only for dietary supplements, but in market areas such as animal feed, health foods, pharmaceutical precursors, artificial sweeteners, and cosmetics as well. The most important role of essential amino acids can be described as [73,74,75]:

- Histidine is an essential amino acid involved in various metabolic pathways and protein synthesis;
- Lysine is an essential amino acid required for protein synthesis and growth in many microorganisms;
- Isoleucine, Leucine, and Valine (Branched-chain amino acids, or BCAAs) are essential amino acids that are important for protein synthesis and energy metabolism. L-valine and L-isoleucine, are frequently utilized as fitness supplements and for individuals with hepatic encephalopathy;
- Methionine is an essential amino acid that is important for protein synthesis, methylation reactions, and sulfur metabolism;
- Phenylalanine is an essential amino acid that serves as a precursor for the synthesis of other important molecules, such as tyrosine and various neurotransmitters;
- Threonine is an essential amino acid that plays a crucial role in protein synthesis and the maintenance of healthy cells;
- Tryptophan is an essential amino acid needed for protein synthesis and various physiological processes.

Microbial production of amino acids has gained significant attention and commercial application due to its efficiency and sustainability compared to traditional chemical synthesis methods. Amino acid synthesis by microorganisms is either fermentative or enzymatic. Amino acids are sometimes produced in quantities of several million tons per year. Microorganisms currently employed for the industrial production of amino acids were created by selection or “brute force” screening procedures after mutation programs. This method of strain enhancement has been surprisingly effective in creating organisms that produce levels of amino acids that are important to industry [76].

Methionine, threonine, and isoleucine are also produced by a branching process in bacteria that also produces lysine. *Escherichia coli* is one example of an organism that closely regulates this mechanism [77]. Microorganisms such as *Corynebacterium glutamicum*-gram-positive bacterium, through genetic–metabolic engineering is commonly used in industrial fermentation processes for the large-scale production of the amino acid L-lysine (member of the aspartate family of amino acids), but also l-glutamate family amino acids (GFAAs) (l-glutamate, l-arginine, l-citrulline, l-ornithine, l-proline, l-hydroxyproline, γ-aminobutyric acid, and 5-aminolevulinic acid) [76,77,78]. By comparing a high generating strain with the wild-type strain, genome-based strain reconstruction was employed to improve the lysine production rate of *C. glutamicum* [79]. L-glutamate is the most important amino acid produced followed by L-lysine.

Large-scale fermentation processes are employed to cultivate the bacterium under controlled conditions, providing it with the necessary nutrients and optimal growth environment. As the bacterium grows and metabolizes the carbon source, it produces and excretes amino acids into the fermentation broth. The accumulated amino acids can then be isolated and purified for various uses, including food additives, pharmaceuticals, and other industrial applications [80,81]. The specific extraction methods can vary depending on the type of bacteria, the amino acids of interest, and the desired level of purity. Optimization of the extraction protocol is often required to achieve the best results for a particular application. Amino acids can be extracted from bacteria using various commonly used techniques [80,81,82,83,84,85,86,87,88,89,90,91,92,93,94]:

(a) Cell Disruption: The first step in extracting amino acids from bacteria is to disrupt the bacterial cells to release their contents. This can be achieved through mechanical disruption methods such as sonication (using ultrasonic waves), homogenization (using a blender or homogenizer), or bead beating (using glass or ceramic beads). These methods break the cell walls and release the intracellular components, including amino acids.
(b) Filtration: After cell disruption, the resulting mixture needs to be separated to remove the cell debris and other large particles. Filtration techniques, such as centrifugation or microfiltration, can be employed to separate the liquid phase containing the extracted amino acids from the solid residue.
(c) Precipitation: To concentrate and separate the amino acids from the liquid extract, precipitation methods can be used. The most common approach is to adjust the pH of the solution to the isoelectric point of the amino acids of interest, causing them to become insoluble and precipitate out. The precipitated amino acids can then be separated by centrifugation or filtration.
(d) Chromatography: Chromatography techniques, such as ion exchange chromatography or high-performance liquid chromatography (HPLC), are often employed for further purification and separation of specific amino acids. These methods utilize the differences in charge, size, or hydrophobicity of the amino acids to separate them into individual components.
(e) Desalting: If the extracted amino acids contain high levels of salts or other impurities, desalting steps may be necessary. It can be achieved by dialysis or using desalting columns, which remove the salts and other small molecules, leaving behind purified amino acids.

Several microorganisms have been used in the production of L-threonine. The synthesis of acetate limits *E. coli*’s production of L-threonine. Transductional crosses were used in *Serratia marcescens* to create a high threonine production by combining numerous feedback control mutations into one organism [82,83].

L-leucine production was reported in analogue-resistant mutants isolated from the glutamate-producing *Bacillus* (*Brevibacterium*) *lactofermentum* through random mutagenesis and then refined through additional mutagenesis stages or by applying metabolic engineering approaches to *C. glutamicum* [84,85].

Isoleucine production can be completed by fermentation with various bacteria such as classically derived mutants of *C. glutamicum*, *S. marcescens*, *C. glutamicum* ssp. *flavum* [85,86]. Valine, branched-chain amino acids, is used in the production of various chemicals. It is produced by engineered bacteria such as *C. glutamicum* or *E. coli* [87]. Engineered wild-type strain *E. coli* is able to overproduce histidine [88,89]. Methionine production has been achieved in genetically modified *E. coli* bacteria as well [90]. Phenylalanine, an aromatic amino acid, is used as well as a flavoring agent, and it can be produced by bacteria like *E. coli* or *C. glutamicum* [91]. Threonine production has been improved in engineered *E. coli* [92]. Tryptophan can be produced by bacteria such as *E. coli* or *C. glutamicum* [93,94].

### 3.2. Microbial Production of Essential Vitamins

Certain strains of bacteria or yeast can produce vitamins in large quantities. The manufacture of vitamins is another biotechnological approach. This approach is commonly used in industrial processes to produce vitamins that are used as dietary supplements, food additives, or in the pharmaceutical industry [95]. Like the essential amino acids, the vitamins needed by humans must also be taken in with food. The majority of vitamins are generated chemically in industry, while biotechnological methods are mostly used to produce vitamins. Dietary supplements fortified with these microbial-derived nutrients can help meet dietary requirements, particularly in individuals with specific dietary restrictions or deficiencies. Vitamins are essential for various physiological processes in the human body. These vitamins can be incorporated into food supplementation formulations to address specific nutrient deficiencies [96]. Compared to essential amino acids, the annual quantities produced are much smaller. However, the prices for the vitamins produced are significantly higher [96,97]. Vitamin extraction from microorganisms involves different processes [96,97,98,99,100,101,102,103,104,105,106,107]:

(a) Cell disruption: Break open the bacterial cells to release the intracellular contents, including the vitamin. Cell disruption methods can include mechanical disruption (e.g., bead milling, high-pressure homogenization) or enzymatic treatments.
(b) Filtration and clarification: After cell disruption, the resulting mixture is typically filtered to remove large debris and cell fragments. Further clarification steps, such as centrifugation or filtration through membranes, can be performed to obtain a clearer solution.
(c) Purification: Purify the extracted vitamin from other cellular components and impurities. Various techniques can be employed, such as chromatography (e.g., column chromatography, high-performance liquid chromatography) or crystallization, depending on the specific vitamin and its properties.
(d) Concentration and drying: Concentrate the purified vitamin solution to increase its potency. This can be performed through techniques like evaporation or freeze-drying (lyophilization) to remove the solvent and obtain a dry, stable vitamin powder.
(e) Quality control: Perform rigorous quality control tests on the extracted and purified vitamin to ensure its potency, purity, and safety. These tests may include assays for vitamin content, impurity analysis, and microbial testing.

Of the 13 human vitamins, the following ones are produced via microorganisms: vitamin B2 (riboflavin) by either the fungus *Ashbya gossypii* or the bacterium *Bacillus subtilis*, and vitamin B12 with strains of *Pseudomonas denitrificans* or *Propionibacterium freundenreichii*, other possible producers, such as *Sinorhizobium meliloti*, different *Lactobacillus* strains and even *E. coli* [98,99,100,101,102].

Vitamin D, also known as cholecalciferol, can be synthesized by certain strains of yeast. *Saccharomyces cerevisiae* is commonly used in the industrial production of vitamin D. The yeast is genetically modified to produce a specific enzyme that enables the conversion of a precursor molecule into vitamin D [97,103]. Vitamin C, also known as ascorbic acid, can be produced through microbial fermentation. Several microorganisms, including certain strains of bacteria and yeast, are capable of synthesizing vitamin C. The most commonly used microorganism for large-scale production is *Gluconobacter oxydans* and *Ketogulonicigenium vulgare* [104]. Certain strains of bacteria, such as *Bacillus subtilis*, are used for the production of vitamin K2 [105,106,107].

*P. freudenreichii* has the capacity to synthesize other compounds, mostly propionic acid and trehalose besides vitamin B12, and this aspect can also boost its industrial appeal [108]. These are just a few examples of how microorganisms can be used to produce vitamins. The specific microbial strains, growth conditions, and fermentation processes may vary depending on the desired vitamin and the industry requirements. Microbial production of vitamins offers a sustainable and cost-effective alternative to chemical synthesis methods and helps meet the demand for these essential nutrients.

### 3.3. Microbial Production of Functional Compounds

A highly topical field of research in biotechnology is the microbial production of natural substances, many of which have health-promoting effects or are used for therapeutic purposes. Both microorganisms themselves such as bacteria, fungi, algae, and plants synthesize natural substances [109,110]. In the case of the latter, it is assumed that there are about 200,000 different natural substances. These compounds can include secondary metabolites, enzymes, and other bioactive substances. Microbial natural products often contain bioactive compounds with specific functional properties. These may include antioxidant, antimicrobial, anti-inflammatory, immune-modulating, or cholesterol-lowering effects. Incorporating these compounds into dietary supplements can offer targeted health benefits and support various aspects of human health [111].

Certain microbial metabolites possess antioxidant properties. Microorganisms can produce a wide range of antioxidants, such as phenolic compounds, flavonoids, and carotenoids. These compounds can help reduce oxidative stress in the body, protect cells from damage, and potentially prevent chronic diseases. Antioxidant-rich microbial natural products can be incorporated into dietary supplements or functional foods to enhance their nutritional value and provide antioxidant benefits [69,112].

While plants are traditionally known as the primary source of phytochemicals, scientists have discovered that many microorganisms such as bacteria, fungi, and algae are also capable of synthesizing these compounds [113]. Microorganisms produce phytochemicals as part of their natural metabolic processes. These compounds serve various functions for microorganisms, including defense against pathogens, competition for resources, and communication within microbial communities. Some microorganisms produce phytochemicals that have antimicrobial, antioxidant, anticancer, or other health-promoting properties. Several microbial metabolites have been identified as antioxidants and have shown potential in scavenging ROS and free radicals. These compounds can donate electrons or hydrogen atoms to neutralize free radicals and prevent oxidative damage. Furthermore, some microbial metabolites, such as certain peptides and polyphenols, have been reported to possess antioxidant properties and can inhibit oxidative processes by various mechanisms, including metal chelation, radical scavenging, and modulation of cellular antioxidant enzymes [114,115]. It is important to note that the antioxidant activity of microbial metabolites can vary depending on the specific compound, concentration, and experimental conditions. Additionally, their bioavailability and effectiveness in the human body can also be influenced by factors such as absorption, metabolism, and distribution.

Overall, microbial metabolites can serve as a valuable source of natural antioxidants, and their potential applications in medicine, food preservation, and nutraceutical industries are actively being explored. A few examples of microbial production strategies for phenolics include:

- Bacterial production: some bacteria have the ability to produce phenolic compounds through their metabolic pathways. For instance, *E. coli* has been engineered to produce various phenolics, including resveratrol [116], a well-known antioxidant compound found in grapes and red wine. It was initially believed that resveratrol was only produced by plants, but later studies revealed that certain species of bacteria and fungi can also synthesize this compound (e.g., *Saccharomyces cerevisiae*) [117,118,119]. By introducing specific genes encoding enzymes involved in phenolic biosynthesis, researchers have successfully developed bacterial strains capable of producing these compounds;
- Yeast production: certain strains of yeast, such as *Saccharomyces cerevisiae*, have been engineered to produce phenolic compounds [117,118,119]. Through genetic modifications, key enzymes involved in the phenolic biosynthetic pathway can be introduced into the yeast, enabling them to convert simple precursors into desired phenolic compounds. This approach has been employed to produce compounds like vanillin, which is a commonly used flavoring agent [120,121];
- Fungal production: fungi are also a promising source for phenolic compound production. Filamentous fungi, such as *Aspergillus*, *Rhizopus* and *Penicillium* species, have been studied for their ability to produce phenolics [122], like tannins and coumarins, lignin derivatives, and flavonoids like naringenin and apigenin [123].

Optimization of culture conditions and genetic engineering approaches can be employed to enhance phenolic production in these fungi. While microbial production of phenolics holds promise, the field is still under active research, and further optimization and development are needed to enhance production yields and commercial viability [124].

Carotenoids are pigments responsible for the bright colors of fruits and vegetables. Some microorganisms, like certain strains of bacteria and fungi, produce carotenoids. In terms of economic production of—especially natural—pigments, ways of biotechnological production, specifically the production of pigments with various microorganisms, have recently been explored as an alternative to traditional methods, such as extraction from plants or chemical synthesis [125,126,127,128,129]. They are widely used in various industries, including food, pharmaceuticals, and cosmetics, due to their antioxidant and provitamin A activities.

Several microorganisms have been explored for their ability to produce carotenoids, including bacteria and fungi:

- *Escherichia coli*: Genetic engineering techniques have been used to introduce carotenoid biosynthetic pathways into *E. coli*, enabling the production of carotenoids such as β-carotene and lycopene. [130];
- *Agrobacterium* sp.: The bacterium has been engineered to produce astaxanthin, a highly valued carotenoid pigment, through the introduction of genes from other carotenoid-producing organisms. [131];
- *Paracoccus* sp.: Some species of *Paracoccus* (e.g., *Paracoccus carotinifaciens*) have been found to naturally produce carotenoids such as canthaxanthin and astaxanthin [132];
- *Blakeslea trispora* and *Phycomyces blakesleeanus*: commercially used for the production of β-carotene. It can be cultured under controlled conditions, and its carotenoid content can be enhanced by optimizing growth parameters. [133,134];
- *Neurospora crassa*: Genetic engineering approaches have been applied to *N. crassa* to enhance its carotenoid production capacity. [135];
- *Phycomyces blakesleeanus*: This fungus naturally produces β-carotene and can be cultivated to enhance carotenoid yields. [136]

Microorganisms can already produce astaxanthin in food purity are as well *Xanthophyllomyces dendrorhous*, *Brevibacterium* sp., *Puccinia graminis* and *Sphingomonas astaxanthinbreifaciens* [132,137]. Carotenoid’s production is already possible with algae and in filamentous fungi (especially *Monascus* species) and can be produced on a large industrial scale. Continuous advancements in biotechnology and synthetic biology are continually expanding the possibilities for microbiological production of carotenoids, offering sustainable and efficient alternatives to traditional methods [138]. The followings steps are involved in the carotenoid’s extraction process from microorganisms [138,139,140,141,142,143,144,145,146]:

(a) Cell Disruption: Break open the harvested cells to release the carotenoids. Cell disruption methods can include physical techniques like sonication (ultrasonication), high-pressure homogenization, or mechanical methods such as bead milling or grinding. The objective is to rupture the cell walls and release the intracellular contents.
(b) Extraction: Extract the carotenoids from the disrupted cells using an appropriate solvent. Common solvents for carotenoid extraction include organic solvents like acetone, ethyl acetate, or hexane. The choice of solvent depends on the nature of the carotenoids and their solubility properties.
(c) Separation and Purification: After extraction, the crude extract is obtained, which may contain impurities and other unwanted compounds. Purify the carotenoid extract using techniques such as liquid–liquid extraction, chromatography (e.g., column chromatography or high-performance liquid chromatography), or filtration methods to obtain a pure carotenoid fraction.
(d) Concentration and Drying: Concentrate the purified carotenoid solution using techniques such as rotary evaporation or nitrogen gas blowdown. Finally, dry the concentrated carotenoid solution to remove any residual solvent and obtain a dry carotenoid powder.

The specific extraction protocol may vary depending on the microorganism, the targeted carotenoids, and the intended application of the extracted pigments. Optimization of the extraction process can involve multiple experimental iterations to achieve the highest yield and purity of carotenoids.

The ability of microorganisms to synthesize such compounds is of great interest in fields such as medicine, agriculture, and biotechnology. The discovery of phytochemical production in microorganisms has expanded the potential sources of these bioactive compounds. Scientists are exploring the microbial world to identify new strains capable of producing valuable phytochemicals for various applications, including medicine, agriculture, and food production [139].

Microbes are often employed in industrial fermentation processes to produce enzymes that can be used in food supplementation. Enzymes like proteases (produced by *Bacillus subtilis* or *Aspergillus oryzae*) [140], lipases (derived from *Candida rugosa* or *Rhizopus oryzae*) [141], cellulases (derived from *Trichoderma reesei* or *Aspergillus niger*, *Pseudomonas fluorescens*, *Bacillus subtilIs*, *E. coli*, Serratia marcescens) [142], pectinases (produced by *Aspergillus* spp., *Erwinia* spp., *Penicillium* spp., *Bacillus* spp., *Trichoderma viride*, *Mucor piriformis*, *Yarrowia lipolytica*, *Schizophyllum commune*, *Saccharomyces* spp., *Torulopsis kefyr*, *Candida pseudotropicalis* spp.) [143], and amylases (produced by *Bacillus licheniformis* or *Bacillus amyloliquefaciens*) [144], can aid in the digestion and absorption of proteins, fats, and carbohydrates, respectively, and are used in various industries, including the production of biofuels, food processing, and textile manufacturing [145,146].

Overall, microorganisms have the capability to produce a wide range of natural plant substances, and their study and utilization hold great potential in fields such as medicine, agriculture, and biotechnology. Access to plant phytochemicals is problematic because isolation from the plant is very laborious due to the often-low concentration and the presence of many other natural substances, and chemical synthesis is not possible or unprofitable in many cases. Therefore, microbial production of plant-derived natural products is considered a promising alternative. In this process, the genes for plant biosynthetic pathways are expressed in suitable microorganisms.

## 4. Future Considerations

### 4.1. Aspects Related to Microbial Production

Advantages of microbial production of secondary metabolites for dietary supplements formulations include the ability to use renewable feedstocks, high production yields, and the potential for cost-effective large-scale production. Additionally, it can be more environmentally friendly compared to chemical synthesis, as it reduces the reliance on petrochemicals and minimizes waste generation [147,148,149]. As a general overview of the microbial production process for such metabolites, must be taken into consideration several aspects:

- Selection of microorganism: Various microorganisms have been engineered or naturally occur with the ability to produce specific metabolites. Researchers select the appropriate microorganism based on its natural capabilities or modify its genetic makeup through genetic engineering techniques to enhance targeted metabolites production;
- Substrate selection: Microorganisms require a carbon source for growth and metabolites production. Common substrates include glucose, molasses, starch, or other renewable biomass sources. The choice of substrate depends on the target metabolite and the cost-effectiveness of the production process;
- Optimization of culture conditions: Microorganisms need specific environmental conditions to thrive and produce metabolites efficiently. Factors such as temperature, pH, oxygen levels, and nutrient availability are optimized to create an ideal growth environment for the microorganism;
- Genetic Engineering: Genetic engineering techniques are employed to modify the metabolic pathways of microorganisms. This can involve introducing or overexpressing genes related to the metabolite biosynthesis pathway, removing competing pathways, or enhancing precursor supply. These modifications aim to increase the yield and productivity of the desired metabolite;
- Fermentation process: The selected microorganism is cultured in a large-scale fermentation process. Fermenters provide controlled conditions for the microorganisms to grow and produce metabolites. The process typically involves batch, fed-batch, or continuous fermentation modes, depending on the specific requirements of the microorganism and the desired metabolite production;
- Downstream processing: Once the fermentation process is complete, the metabolites need to be separated and purified from the fermentation broth. This process usually includes steps such as cell removal, filtration, precipitation, chromatography, and drying. The purification steps ensure the final product meets the required quality standards.

Advantages of microbial production of metabolites include the ability to use renewable feedstocks, high production yields, and the potential for cost-effective large-scale production. Additionally, this method can be more environmentally friendly compared to chemical synthesis, as it reduces the reliance on petrochemicals and minimizes waste generation [147].

A final essential point when considering the biodiversity of microorganisms is the fact that a large proportion of the bacteria and archaea found on Earth have never been studied before. Scientists also speak here of the so-called “microbial dark matter”. With the help of the DNA and RNA sequencing technologies available today, it has already been shown that the microbiomes of many habitats on Earth are dominated by previously uncultivated bacteria and archaea [148]. The metabolic performance of these microorganisms can so far only be predicted from the genome sequences. However, it is expected that with the help of novel cultivation approaches as well as synthetic biology methods, the metabolic capabilities of these organisms will be better researched in the future and made usable for biotechnology.

A recent trend is biotechnology from the ocean. With this respect, metabolic engineering of microorganisms is checked for development of cell factories and bioprocesses to use sustainable marine algae extracts as nutrients for further production of value-added products. Traditional biotechnology raw materials are replaced by marine macroalgae, due to their ingredients and are considered to be the latest generation of renewable raw materials [115,149].

### 4.2. Aspects Related to Consumers Acceptance

**Figure 3:**
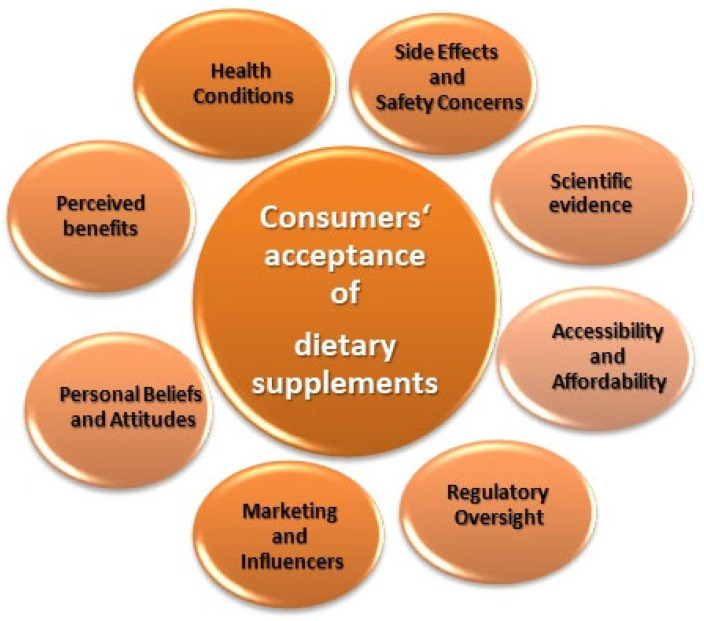
Factors influencing consumers’ acceptance of dietary supplementation.

In general, consumers’ acceptance of dietary supplementation derived from microbial production can vary depending on various factors such as health conditions, scientific evidence, benefits that consumers perceive, personal beliefs and attitude, marketing influence, accessibility and affordability, and regulatory oversight [150,151,152] (Figure 3). Furthermore, consumer demand for sustainable and environmentally friendly products has led to the exploration of microbial production methods as alternatives to traditional manufacturing.

Consumers are more likely to accept dietary supplementation if they perceive it to provide tangible health benefits. This can include improving overall well-being, addressing specific nutrient deficiencies, supporting immune function, enhancing athletic performance, or promoting specific health outcomes like better skin or hair health.

Strong scientific evidence supporting the effectiveness and safety of a particular dietary supplement increases consumer acceptance. Studies published in reputable scientific journals, clinical trials, and expert recommendations can influence consumer perception and build trust.

Consumers with specific health conditions or deficiencies may be more inclined to accept dietary supplementation as a means to address those issues. For example, individuals with diagnosed nutrient deficiencies or certain medical conditions may be more open to taking supplements recommended by healthcare professionals.

Clever marketing strategies and endorsements from influencers or celebrities can significantly impact consumer acceptance of dietary supplements. Effective marketing campaigns that highlight potential benefits, target specific consumer groups, and promote trustworthiness and reliability can influence consumer choices.

The presence of robust regulatory oversight and quality control measures instills confidence in consumers. Regulatory bodies, such as the U.S. Food and Drug Administration (FDA) and the European Food Safety Authority (EFSA), play a crucial role in evaluating the safety, efficacy, and labeling of dietary supplements. Consumers are more likely to accept supplements that comply with such regulations and are backed by appropriate certifications.

Personal beliefs, cultural factors, and attitudes towards health and wellness can influence consumer acceptance of dietary supplementation. Some individuals may have a proactive approach to health and be more open to incorporating supplements into their routines, while others may prefer obtaining nutrients solely from whole foods.

Concerns about potential side effects and safety issues can impact consumer acceptance. Consumers are more likely to accept supplements with minimal reported side effects, well-established safety profiles, and clear dosage instructions.

The availability and affordability of dietary supplements can also affect consumer acceptance. The affordability of microbial products is an important factor influencing consumer acceptance. If supplements are readily accessible and reasonably priced, consumers may be more willing to incorporate them into their daily routines.

Ultimately, consumers’ acceptance of microbial products often depends on their taste, texture, and overall quality. Products that deliver a positive sensory experience, comparable to or better than conventional alternatives, are more likely to be embraced by consumers.

It’s important for consumers to make informed decisions about dietary supplementation and consult healthcare professionals before starting any new supplement regimen, particularly if they have pre-existing health conditions or are taking medications that could interact with the supplements. In addition, it is important to know that consumer acceptance can vary across different regions and demographics [4,153,154,155,156,157]. Conducting market research, understanding consumer preferences, and engaging in continuous dialogue with consumers can help companies gauge and respond to consumer acceptance of microbial products in general. Providing clear and accurate information about the science behind microbial production can help address any concerns or misconceptions consumers may have.

### 4.3. Achievements versus Limitations

Microbial-produced dietary supplements have gained significant attention due to their potential to provide health benefits. There are some existing achievements and limitations associated with microbial-produced dietary supplements. Certain microbes have the ability to produce essential nutrients such as vitamins and amino acids. Microbial-produced dietary supplements can provide a source of these vital nutrients, especially for individuals with dietary restrictions or deficiencies. While microbial-produced dietary supplements hold promise, more research is needed to understand their mechanisms of action, optimal dosages, and potential interactions with medications or existing health conditions. In addition, there are some other limitations:

- Standardization and quality control: Ensuring consistent and reliable production of microbial-produced dietary supplements can be challenging. Variability in microbial strains, fermentation processes, and product formulation can affect the quality and efficacy of the supplements. Strict quality control measures are necessary to ensure safety and effectiveness;
- Shelf stability: Some microbial-produced dietary supplements may have limited shelf stability due to the presence of live microorganisms. These products require proper storage and handling conditions to maintain viability and efficacy. Additionally, some supplements may require refrigeration, which can limit their accessibility and convenience;
- Efficacy and individual variation: The efficacy of microbial-produced dietary supplements can vary among individuals. Factors such as the existing gut microbiota composition, overall health status, and individual response can influence the effectiveness of these supplements. What works for one person may not work the same way for another;
- Regulatory considerations: The regulatory landscape for microbial-produced dietary supplements can be complex and varies across countries. Ensuring compliance with regulations and standards can be challenging for manufacturers, and the lack of standardized guidelines can create uncertainties in the industry.

## 5. Conclusions

Microbial production can provide solutions such as plant-based ingredients, alternative sweeteners, and vitamins, which can appeal to health-conscious and environmentally aware consumers. Modern process technology is utilized to control industrial production processes in the bioreactor in the best possible way. Currently, quality inspection of batches normally occurs only after production, which takes time and frequently results in significant losses. Biotechnologists want to be able to control and monitor the quality of bioproduction beginning with the initial stages of production. Genetic engineering might be needed to get over several bottlenecks, which might not be well welcomed by the general public, especially vegans and vegetarians who are highly worried about their dietary choices and the use of engineered microorganisms. While microbial natural compounds hold promise, further research is needed to fully understand their mechanisms of action, optimize their therapeutic potential, and ensure their safety and efficacy. When formulating food supplementation products, it’s crucial to consider the specific health benefits, stability, and safety of the microbial metabolites being used. Additionally, appropriate quality control measures should be implemented to ensure the consistency and efficacy of the final product. Researchers are actively investigating the potential dietary and nutraceutical applications of different microbial compounds for gut-related disorders. Microbial production offers unique advantages in terms of sustainability, efficiency, and versatility. Educating consumers about the benefits, safety, and production methods of microbial products is crucial.
